# Chemical Constituents of Malaysian *U. cordata* var. *ferruginea* and Their *in Vitro* α-Glucosidase Inhibitory Activities

**DOI:** 10.3390/molecules21050525

**Published:** 2016-04-27

**Authors:** Nur Hakimah Abdullah, Fatimah Salim, Rohaya Ahmad

**Affiliations:** 1 Faculty of Applied Sciences, Universiti Teknologi MARA, 40450 Shah Alam, Selangor Darul Ehsan, Malaysia; kimsyaz@yahoo.com; 2 Center of Foundation Studies, Universiti Teknologi MARA, Dengkil Campus, 43800 Dengkil, Selangor Darul Ehsan, Malaysia; fatimahsalim10@gmail.com; 3 Atta-ur-Rahman Institute for Natural Products Discovery, Universiti Teknologi MARA, 42300 Bandar Puncak Alam, Selangor Darul Ehsan, Malaysia

**Keywords:** *Uncaria cordata*, phytochemistry, α-glucosidase inhibition, 2-hydroxybenzoic acid, 2,4-dihydroxybenzoic acid, loganin

## Abstract

Continuing our interest in the *Uncaria* genus, the phytochemistry and the *in-vitro* α*-*glucosidase inhibitory activities of Malaysian *Uncaria cordata* var. *ferruginea* were investigated. The phytochemical study of this plant, which employed various chromatographic techniques including recycling preparative HPLC, led to the isolation of ten compounds with diverse structures comprising three phenolic acids, two coumarins, three flavonoids, a terpene and an iridoid glycoside. These constituents were identified as 2-hydroxybenzoic acid or salicylic acid (**1**), 2,4-dihydroxybenzoic acid (**2**), 3,4-dihydroxybenzoic acid (**3**), scopoletin or 7-hydroxy-6-methoxy-coumarin (**4**), 3,4-dihydroxy-7-methoxycoumarin (**5**), quercetin (**6**), kaempferol (**7**), taxifolin (**8**), loganin (**9**) and β-sitosterol (**10**). Structure elucidation of the compounds was accomplished with the aid of 1D and 2D Nuclear Magnetic Resonance (NMR) spectral data and Ultraviolet-Visible (UV-Vis), Fourier Transform Infrared (FTIR) spectroscopy and mass spectrometry (MS). In the α-glucosidase inhibitory assay, the crude methanolic extract of the stems of the plant and its acetone fraction exhibited strong α-glucosidase inhibition activity of 87.7% and 89.2%, respectively, while its DCM fraction exhibited only moderate inhibition (75.3%) at a concentration of 1 mg/mL. The IC_50_ values of both fractions were found to be significantly lower than the standard acarbose suggesting the presence of potential α-glucosidase inhibitors. Selected compounds isolated from the active fractions were then subjected to α-glucosidase assay in which 2,4-dihydroxybenzoic acid and quercetin showed strong inhibitory effects against the enzyme with IC_50_ values of 549 and 556 μg/mL compared to acarbose (IC_50_ 580 μg/mL) while loganin and scopoletin only showed weak α-glucosidase inhibition of 44.9% and 34.5%, respectively. This is the first report of the isolation of 2-hydroxybenzoic acid, 2,4-dihydroxybenzoic acid and loganin from the genus and the first report of the α-glucosidase inhibitory potential of 2,4-dihydroxybenzoic acid.

## 1. Introduction

*Uncaria* (Rubiaceae) is amongst the genera known for its alkaloid content. The majority of alkaloids founds in *Uncaria* are of the indole and oxindole type [1]. Continuing our interest in the genus, we have reported the isolation of two new heteroyohimbine-type oxindole alkaloids, namely, rauniticine-*allo*-oxindole B and rauniticinic-*allo* acid B, along with five of their stereoisomers including four pentacyclic oxindole alkaloids, isopteropodine, pteropodine, uncarine F and isopteropodic acid from Malaysian *Uncaria longiflora* var. *pteropoda* [2,3,4]. In contrast, the non-alkaloid constituents of the genus have not been widely reported, although the isolation of flavonoids and other chemical constituents present in *Uncaria* has attracted more interest lately [5]. Flavonoids, terpenes, quinovic acid glycosides, and coumarins have been isolated from *Uncaria.* Recently, we have also reported the isolation of a novel flavonoid (−)-2*R*,3*R*-3,5,4’-trihydoxyflavan-[6,7:5”,6”]-2”-pyranone, named uncariechin, along with (−)-*epi*afzelechin and (−)-*epi*catechin, from the methanol extract of the leaves of *Uncaria longiflora* var. *pteropoda* [6].

Out of 14 species of *Uncaria* available in Malaysia, *Uncaria cordata* var. *ferruginea* is one of the most common representatives. This species of *Uncaria* comprises of two major entities namely, *Uncaria cordata* var. *ferruginea and Uncaria cordata* var. *cordata*. A total of eight alkaloids have been found in *Uncaria cordata*, including dihydrocorynantheine, corynoxine, corynoxine B, 3-*epi*-β-yohimbine, rhynchophylline, isorhyncophylline, uncarine A and uncaric acid D [1]. Throughout the literature, there has only been one report on the phytochemistry of Malaysian *Uncaria cordata* var. *ferruginea* which yielded dihydrocorynantheine from an ethanolic leaf extract of the plant [7]. To date, there have been no other reports on the non-alkaloid constituents and biological activities of the plant. Therefore, in continuation of our interest on the phytochemicals of *Uncaria*, we now report the isolation of non-alkaloidal constituents from Malaysian *Uncaria cordata* var. *ferruginea*.

## 2. Results and Discussion

**Figure 1:**
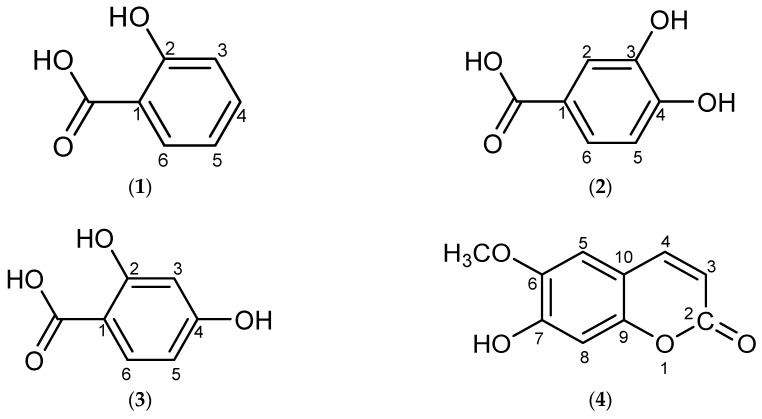

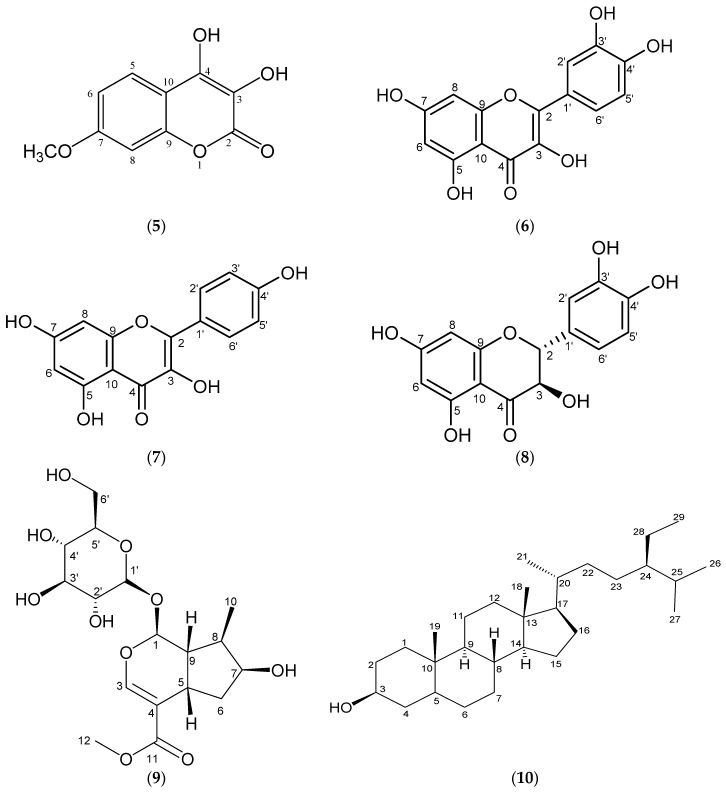
Compounds from *Uncaria cordata* var. *ferruginea*. The constituents were identified as 2-hydroxybenzoic acid (salicylic acid), 2,4-dihydroxybenzoic acid, 3,4-dihydroxybenzoic acid, 7-hydroxy-6-methoxycoumarin (scopoletin), 3,4-dihydroxy-7-methoxycoumarin, quercetin, kaempferol, taxifolin, loganin and β-sitosterol.

Our study of the phytochemistry of Malaysian *Uncaria cordata* var. *ferruginea* led to the isolation and identification of 10 constituents, the structures of which are shown in Figure 1. The compounds 2-hydroxybenzoic acid (**1**), 2,4-dihydroxy-benzoic acid (**2**) and loganin (**9**) were reported for the first time from the genus [5]. In this section, the structure elucidation of these three compounds will be discussed briefly, while the spectral data for the remaining compounds are listed in the Experimental Section.

Compound **1** was isolated as a white amorphous solid with a melting point of 154 °C. Its mass spectrum showed a [M + H]^+^ molecular ion peak at *m*/*z* 139, which corresponds to the molecular formula C_7_H_6_O_3_. The UV spectrum of the compound displayed an absorption peak at 308 nm indicative of an aromatic-type compound due to π → π* transition of benzene chromophore. Its IR spectrum exhibited absorption bands at 3237 cm^−1^ due to a hydroxyl group, 1675 cm^−1^ for a conjugated carbonyl group, 1613 cm^−1^ for aromatic C=C and 759.7 cm^−1^ (C=C-H). The ^1^H-NMR spectrum of this compound showed that all proton signals appeared in the aromatic region confirming the presence of a benzene ring. Two doublet of doublet signals were observed at δ 6.78 (1H, *J* = 7.86, 1.08 Hz, H-3) and δ 6.80 (1H, *J* = 7.20, 1.08 Hz, H-5). A triplet was observed at δ 7.28 (1H, H-4) while a doublet of doublets was observed at δ 7.84 (1H, *J* = 7.68, 1.68 Hz, H-6). The COSY spectrum of compound **1** showed H-H correlations between H-4, H-5 and H-6. However, no hydroxyl peak was observed in the ^1^H-NMR spectrum of compound **1**. This may be due to deuterium exchange of the MeOD with the acidic hydrogen present in the molecule [8]. In the ^13^C-NMR spectrum of compound **1**, the presence of six carbon signals between 100–130 ppm indicated the presence of aromatic ring carbons. One oxyaryl carbon was observed at δ 161.2 (C-2) while one quaternary carbon was seen at δ 118.9 (C-1). The rest of the carbon signals appearing at δ 115.7, 117.5, 130.2 and 132.3 were identified as the C-5, C-3, C-6 and C-4 methine carbons, respectively. Their C-H correlations were established using HMQC and HMBC spectral data. In addition, a very downfield signal at δ 174.8 belonged to the C-7 carboxylate carbonyl function. Based on its 1D and 2D NMR data as well as comparison with literature data [9], compound **1** was determined to be 2-hydroxybenzoic 2-hydroxybenzoic acid or salicylic acid. This is the first report of 2-hydroxybenzoic acid in the genus of *Uncaria*.

Compound **2** was obtained as light yellow crystals. As for compound **1**, the UV spectrum of compound **2** showed a maximum absorption at 298.8 nm indicating an aromatic type compound due to the π → π* transition of a benzene chromophore. The IR spectrum of this compound displayed an absorption pattern similar to that of compound **1** with bands at 3234.24 cm^−1^ (broad, OH), 1674.21 cm^−1^ (C=O carboxylate), 1602.21 cm^−1^ (C=C) and 764.7 cm^−1^ (C=C-H) [10]. Its ^1^H-NMR spectrum displayed the presence of three aromatic proton signals suggesting that the other three positions of a benzene ring might be substituted. In its ^13^C-NMR spectrum, a very downfield signal indicated the presence of a carboxylate carbon while two oxyaryl carbons suggested a dihydroxy-substituted benzoic acid. According to the position of protons and its HMBC and COSY correlations, the three proton signals at δ 6.82 (1H, d, *J* = 8.10 Hz), 7.44 (1H, d, *J* = 1.98 Hz) and 7.45 (1H, dd, *J* = 4.38, 1.92 Hz) were unambiguously assigned as H-6, H-3 and H-5, respectively. As for compound **1**, no OH signals were observed in the ^1^H-NMR spectrum due to deuterium exchange. In its ^13^C-NMR spectrum, six carbon signals were observed at δ 114–150, indicating the presence of an aromatic ring. Two oxyaryl carbons appeared at δ 144.7 (C-2) and δ 150.1 (C-4) while one quaternary carbon was observed at δ 121.8 (C-1). The rest of the carbon signals belonged to methine carbons while a very downfield signal at δ 168.9 was assigned to the carbonyl function of a carboxylate function at C-7. Therefore, by careful assignments of 1D and 2D NMR data, compound **2** was characterized as 2,4-dihydroxybenzoic acid.

Compound **9** was isolated as a colourless oil and its mass spectrum gave an [M + H_2_O]^+^ peak at *m/z* 408 which corresponded to the molecular formula C_17_H_26_O_10_. Its UV spectrum displayed absorption bands at 295 and 330 nm, while the IR spectrum exhibited typical absorption frequencies at 3368, 1655, 1420, 1449 and 669 cm^−1^ for an iridoid-type compound [11]. In the low field region of its ^1^H-NMR spectrum, a proton singlet at δ 7.41 correlating with the signal at δ 150.73 in the ^13^C-NMR spectra as determined by the HMQC experiment was due to the presence of an olefinic proton at position H-3. There were also a pair of doublet of doublets signals appearing at δ 5.30 (d, *J* = 4.5 Hz, 1H) and δ 4.67 (d, *J* = 7.92 Hz, 1H) and assigned as H-1 and H-1’, respectively. In the high field region of the spectrum, a three-proton doublet appearing at δ 1.11 correlating with the signal at δ 12.01 in the ^13^C-NMR was assigned as the methyl protons, C10-H3 while a three-proton singlet observed at 3.70 was attributed to the carbomethoxy group. Four multiplet proton signals at δ 1.64, 1.89, 2.05 and 2.25 were assigned as H-6ax, H-8, H-9 and H-6eq, respectively, while all proton signals observed in range δ 3.00 to 4.00 belonged to protons in the glucose moiety. The ^13^C-NMR spectrum of compound **9** displayed a total of 17 carbon signals. Two carbon signals at δ 41.3 (C-6) and δ 61.36 (C-6)’ representing methylene carbons were identified using the DEPT135 spectrum. The methoxy and methyl carbon signals appeared at δ 50.25 and δ 12.01, while two quaternary carbon signals observed in downfield region at δ 168.19 and 112.64 were assigned as C-11 and C-4. Altogether, there were seven methine carbon signals, two quaternary carbons, one methoxy carbon and one methyl carbon identified with the aid of DEPT 135 and DEPT 90 spectral data. The rest of the carbon signals belonged to hydroxymethine carbons. Finally, an unambiguous assignment of all of the proton and carbon signals using HMQC, HMBC and COSY as well as by comparison with the literature data [9] confirmed that compound **9** was the iridoid glycoside loganin. Other known compounds were characterized by NMR and mass spectroscopy as well as comparison with literature. The structure of these compounds are shown in Figure 1.

### α-Glucosidase Inhibitory Activities

A preliminary screening of α-glucosidase enzyme inhibitory activities was conducted on the crude stem extract, acetone and DCM fractions and four isolated compounds with the aim of identifying potential α-glucosidase inhibitors. The methanolic stem extract of Malaysian *Uncaria cordata* var. *ferrugenia* exhibited a high percentage of α-glucosidase inhibition (87.7%) which is consistent with the results reported in an earlier study [12]. The acetone fraction exhibited strong inhibition (89.2%) while the DCM fraction demonstrated moderate inhibition (75.3%). Generally, plant fractions of different polarity demonstrate different degrees of inhibition effects against α-glucosidase enzyme due to the presence of different phytochemicals [13,14]. The IC_50_ values of both fractions were found to be much lower than the standard acarbose, suggesting the presence of potential α-glucosidase inhibitors, as shown in Table 1. By using a bioassay-guided approach, compounds with α-glucosidase inhibitory properties are more easily targeted from active fractions. The compounds which were available in sufficient quantities including 2,4-dihydroxybenzoic acid (2,4-DHBA), quercetin and loganin isolated from the acetone fraction were assayed against α-glucosidase enzyme. Due to their limited amount, 2-hydroxybenzoic acid, kaempferol and taxifolin could not be subjected to the same assay. 2,4-DHBA and quercetin showed strong inhibitory effect against the α-glucosidase enzyme with IC_50_ values 549 μg/mL (or 3.56 mM) and 556 μg/mL (or 1.84 mM), respectively, compared to the standard acarbose (IC_50_ 580 μg/mL or 0.89 mM). Both compounds showed competitive inhibitory profile for α-glucosidase activity against acarbose despite exhibiting lower activities at molar concentration units. There is sufficient literature to support quercetin as a potential α-glucosidase inhibitor [15,16]. However, to date there has been no specific report on the α-glucosidase inhibitory activity of the benzoic acid derivative 2,4-DHBA. Nevertheless, studies on the α-glucosidase inhibition activity of other phenolic acids such as gallic acid, vanillic acid and hydroxybenzoic acid have found them to demonstrate promising inhibitory effects toward the enzyme. Many studies on this class of compounds have proven their antioxidant, anti-inflammatory, anti-cancer, anti-bacterial as well as anti-hyperglycemic properties [15,16,17,18,19]. Hence the untested compounds from this study may contribute significantly to the activity observed for the acetone fraction.

In this study, the iridoid glycoside loganin was found to exhibit weak α-glucosidase inhibition (44.9%). This weak α-glucosidase inhibitory activity is supported by the work of Hua and coworkers [20] who reported that seven out of fourteen iridoid glycosides isolated from the roots of *Scrophularia ningpoensis* Hemsl. (Scrophulariaceae) demonstrated only moderate α-glucosidase inhibitory activity compared to acarbose (positive control). Similarly, scopoletin, a major compound isolated from the DCM fraction, displayed only weak inhibition (34.5%) against the enzyme. This finding is also supported by earlier studies on selected coumarins (umbelliferone, herniarin, esculetin, isoscopoletin) which showed weak inhibitory activities against α-glucosidase enzyme [21,22]. The α-glucosidase inhibitory activities of fractions and compounds isolated from *Uncaria cordata* var. *ferruginea* are summarized in Table 1. Generally, compounds showing a high percentage of inhibition and low IC_50_ values are considered to possess anti-diabetic activity as they could help in preventing postprandial hyperglycemia by decreasing the rate of carbohydrate degradation to glucose.

## 3. Experimental Section

### 3.1. General Information

TLC and PTLC were performed using pre-coated aluminium-backed supported silica gel 60 F254 (0.2 mm thickness) and glass supported silica gel 60 F254 (0.5 and 1.0 mm thickness). Column chromatography was carried out using silica gel 60, 70–230 mesh ASTM (7734, Merck, Darmstadt, Germany) or Sephadex LH-20 (Sigma-Aldrich, St. Louis, MO, USA). Flavonoids were detected on TLC stained with aluminium chloride (AlCl_3_) reagent. Spots and bands for compounds on TLC, PTLC and radial plates were detected using UV light (254 and 365 nm). Mass spectra were measured on an Agilent 1100 Series Technologies HPLC-TOF LC/MS (Agilent Technologies, Santa Clara, CA, USA). The ultraviolet (UV) spectra were obtained in methanol on a UV-Vis 160i instrument (Shimadzu, Kyoto, Japan). The infrared (IR) data was recorded on a Perkin Elmer Spectrum One FTIR Spectrophotometer as KBr discs (PerkinElmer, Waltham, MA, USA). The ^1^H-NMR and ^13^C-NMR were analyzed on a 600 Ultrashield NMR spectrometer at 600 and 150 MHz or a 500 Ultrashield NMR spectrometer at 500 and 125 MHz or a 300 Ultrashield NMR spectrometer measured at 300 and 75 MHz, respectively (Bruker, Billerica, MA, USA). Chloroform-*d*, acetone-*d*_6_ or methanol-*d*_4_ was used as solvent.

### 3.2. Plant Material

#### 3.2.1. First Batch of Plant

Stems of *Uncaria cordata* var. *ferruginea* was collected from Hutan Pasir Raja, Terengganu in December 2011 and was identified by Dr. Shamsul Khamis (Universiti Putra Malaysia). Flowers of *Uncaria cordata* var. *ferruginea* was collected from Hutan Simpan Endau-Rompin, Pahang in April 2012 and also identified by Dr. Shamsul Khamis. The voucher specimens were deposited at the Herbarium of Institute of Bioscience, Universiti Putra Malaysia.

#### 3.2.2. Second Batch of Plant

Stems of *Uncaria cordata* var. *ferruginea* was collected from Hutan Pasir Raja, Terengganu in September 2013 and was identified by En. Ahmad Zainudin Ibrahim (Universiti Kebangsaan Malaysia). The voucher specimens (HTBP 4318) were deposited at Herbarium Taman Botani Putrajaya, Malaysia.

### 3.3. Extraction and Isolation of Compounds

#### 3.3.1. First Batch of Plant

##### *Stem Extract* 

The DCM extract was subjected to column chromatography for further fractionation using Hex, DCM and MeOH with a gradual increase in solvent polarity to yield a total of 12 fractions. Dark green needles were observed in fraction 2 (F2) and light green needles were observed in fraction 4 (F4). Both fractions were selected for further isolation and purification. These two semi-purified fractions, F2 and F4 were gently washed with a small volume of acetone to afford a colourless crystal triterpene (compound **10**) and light yellow crystals (compound **4**). Based on their TLC profiles, F5 to F9 were pooled and subjected for further purification by glass PTLC using MeOH–CHCl_3_ (3:7) to give a greyish solid (compound **5**).

About 35 g of MeOH extract was subjected to liquid-liquid partitioning using DCM and EtOAc. Upon TLC detection with aluminium chloride reagent, the EtOAc fraction was found to contain flavonoids. This fraction (9.8 g) was further fractionated using a column packed with Sephadex LH-20 employing CHCl_3_–MeOH (9:1) as the solvent system to give four sub-fractions. The TLC profiles of sub-fractions F2 and F4 revealed the presence of flavonoids and repeated column chromatography with Sephadex LH-20 successfully yielded the two flavonols **6** and **7**.

##### *Flower Extract* 

The crude methanolic extract of the flowers (65 g) was subjected to column chromatography using normal phase silica employing DCM, EtOAc and MeOH as solvent system with a gradual increase in solvent polarity to give seven fractions F1–F7. F4 and F5 were pooled and targeted for further purification as they showed a positive flavonoids test. The pooled fraction was then subjected to column chromatography on Sephadex LH-20 using CHCl_3_ and MeOH (9:1) as solvent system to yield pale yellow crystals of compound **8**.

#### 3.3.2. Second Batch of Plant

Successive trituration of the methanolic stems extract of *U. cordata* var. *ferruginea* (296.49 g) with Hex, DCM, acetone and MeOH was carried out in order to overcome the limitations on chromatographic large scale sample introduction. The DCM fraction (11.0 g) was subjected to column chromatography for further fractionation using Hex, DCM and MeOH with a gradual increase in solvent polarity to yield a total of 15 fractions. Based on its TLC profiles observed under UV short wavelength, F12 (3.03 g) was found to contain a potential compound and was subjected to further isolation and purification via column chromatography packed using Sephadex LH-20 with CHCl_3_ and MeOH (9:1) as the solvent system. Out of 12 sub-fractions collected from F12, sub-fractions F9 to F12 were pooled and subjected for further purification by glass PTLC using MeOH–CHCl_3_ (3:7) to give compound **1**.

The acetone fraction (30.52 g) was subjected to liquid-liquid partitioning between MeOH and diethyl ether (DE) to remove polar constituents. The undissolved portion was filtered and the solvent was evaporated off under reduced pressure yielding 14.3 g fraction of DE fraction. Then, the fraction was subjected to column chromatography packed with Sephadex LH-20 using CHCl_3_–MeOH (9:1) as the solvent system to yield a total of forty fractions. The TLC profiles of sub-fractions F6 and F7 revealed potentially interesting compounds and were pooled and rechromatographed with Sephadex LH-20 using CHCl_3_–MeOH (7:3) as the solvent system. Subfractions F1 and F2 were subjected to further isolation and purification with recycling HPLC where isocratic elution employing MeOH and H_2_O (1:1) with UV detection at 254 nm were applied on both fractions. Compounds **2** and **3** were successfully isolated as colorless crystal from F1 while compound **9** was obtained as a colorless oil from F2 by this method.

### 3.4. Physical and Spectral Data of Isolated Compounds

*2-Hydroxybenzoic acid* (**1**). White amorphous solid, wt: 2.3 mg (MeOH), mp 154 °C (mp lit. 153–154 °C). MS *m*/*z* = 139.0537 [M + H]^+^, C_7_H_6_O_3_; UV (MeOH) λ_max_ nm: 308; IR (KBr) υ_max_ cm^−1^: 3237 (OH), 1675 (C=O), 1613 (C=C), 759 (C=C-H); ^1^H-NMR (MeOD, 600 MHz) δ ppm: 7.84 (1H, dd, *J* = 7.68, 1.68 Hz, H-6), 7.28 (1H, t, H-4), 6.78 (1H, dd, *J* = 7.86, 1.08 Hz, H-3), 6.80 (1H, dd, *J* = 7.20, 1.08 Hz, H-5); ^13^C-NMR (MeOD, 150 MHz) δ ppm: 174.8 (C-7), 161.2 (C-2), 132.3 (C-4), 130.2 (C-6), 118.9 (C-1) 117.5 (C-3), 115.7 (C-5) [9].

*2,4-Dihydroxybenzoic acid* (**2**). Light yellow crystals, wt: 3.2 mg (MeOH). UV (MeOH) λ_max_ nm: 298.8; IR (KBr) υ_max_ cm^−1^: 3234 (OH), 1674 (C=O), 1602 (C=C), 764 (C=C-H); ^1^H-NMR (MeOD, 600 MHz) δ ppm: 7.45 (1H, dd, *J* = 4.38, 1.92 Hz, H-5), 7.44 (1H, d, *J* = 1.98 Hz, H-3), 6.82 (1H, d, *J* = 8.10 Hz, H-6); ^13^C-NMR (MeOD, 150 MHz) δ ppm: 168.9 (C-7), 150.1 (C-2), 144.7 (C-4), 122.5 (C-3), 121.8 (C-1), 116.4 (C-5), 114.4 (C-6).

*3,4-Dihydroxybenzoic acid* (**3**). Transparent needles, wt: 1.5 mg (MeOH), mp 198 °C (mp lit. 198–200 °C). MS *m*/*z* = 155. 0494 [M + H]^+^, C_7_H_6_O_4_; UV (MeOH) λ_max_ nm: 293.3; IR υ_max_ cm^−1^: 3280 (OH), 1655 (C=O); ^1^H-NMR (MeOD, 600 MHz) δ ppm: 7.45 (1H, d, *J* = 1.98 Hz, H-2), 7.43 (1H, dd, *J* = 8.22, 2.04, H-6), 6.80 (1H, d, *J* = 8.22 Hz, H-5); ^13^C-NMR (MeOD, 150 MHz) δ ppm: 169.7 (C-7), 149.7 (C-3), 144.5 (C-4), 123.0 (C-1), 122.4 (C-6), 116.4 (C-2), 114.3 (C-5) [23].

*Scopoletin* (**4**). Light yellow crystals, wt: 30.5 mg (DCM), mp 205 °C (mp lit. 204 °C). MS *m*/*z* = 193.0505 [M + H]^+^, C_10_H_8_O_4_; UV (MeOH) λ_max_ nm: 228, 295, 345; IR (KBr) υ_max_ cm^−1^: 3471 (free OH), 2964 (C-H aromatic);^1^H-NMR (CDCl_3_, 600 MHz) δ ppm: 7.63 (1H, d, *J* = 9.48 Hz, H-4), 6.95 (1H, s, H-8), 6.87 (1H, s, H-5), 6.30 (1H, d, *J* = 9.42 Hz, H-3), 6.19 (1H, br s, OH), 3.98 (3H, s, OCH_3_); ^13^C-NMR (CDCl_3_, 150 MHz) δ ppm: 161.5 (C=O), 150.3 (C-6), 149.7 (C-9), 144.0 (C-7), 143.3 (C-4), 113.4 (C-3), 111.5 (C-10), 107.5 (C-5), 103.2 (C-8), 56.4 (OCH_3_) [24].

*3,4-Dihydroxy-7-methoxycoumarin* (**5**). Light grey amorphous solid, wt: 1.5 mg (acetone); ^1^H-NMR (acetone-*d*_6_, 300 MHz) δ ppm: 7.48 (d, 1H, *J* = 2.04 Hz, H8), 7.43 (dd, 1H, *J* = 8.37, 2.19 Hz, H6), 6.88 (d, 1H, *J* = 8.25 Hz, H5), 3.81 (s, 3H, OCH_3_); ^13^C-NMR (Acetone-*d*_6_, 75 MHz) δ ppm: 166.2 (C=O), 150.4 (C-7), 145.1 (C-4), 139.8 (C-9), 138.7 (C-3), 122.3 (C-6), 121.6 (C-10), 116.3 (C-8), 115.0 (C-5), 50.9 (OCH_3_) [25].

*Quercetin* (**6**). Yellow amorphous powder, wt: 3.8 mg (acetone), mp 310–313 °C (mp lit. 313–315 °C). MS *m*/*z* = 303.0678 [M + H]^+^, C_15_H_10_O_7_; UV (MeOH) λ_max_ nm: 295, 338, 385; IR (KBr) υ_max_ cm^−1^: 3411 (OH), 1665 (C=O), 1615 (C=C); ^1^H-NMR (acetone-*d*_6_, 600 MHz) δ ppm : 12.19 (1H, br s, OH), 7.85 (1H, d, *J* = 2.04 Hz), 7.72 (1H, dd, *J* = 8.46, 2.16 Hz), 7.01 (1H, d, *J* = 8.46 Hz) 6.54 (1H, d, 2.04 Hz), 6.28 (1H, d, 2.04 Hz); ^13^C-NMR (Acetone-*d*_6_, 150 MHz) δ ppm: 175.7 (C=O), 164.2 (C-7), 161.5 (C-5), 156.9 (C-9), 147.5 (C-4’), 146.1 (C-2), 145.0 (C-3’), 135.9 (C-3), 122.9 (C-1’), 120.6 (C-6’), 115.3 (C-5’), 114.9 (C-2’), 103.2 (C-10), 98.3 (C-6), 93.6 (C-8) [26].

*Kaempferol* (**7**). Pale yellow amorphous powder, wt: 1.2 mg (acetone), mp 275–277 °C (mp lit. 276–278 °C). MS *m*/*z* = 285.0391 [M − H]^−^, C_15_H_10_O_6_; UV (MeOH) λ_max_ nm: 310, 380; IR (KBr) υ_max_ cm^−1^: 3324 (OH), 1660 (C=O), 1614 (C=C); ^1^H-NMR (MeOD, 500 MHz) δ ppm : 8.09 (2H, dd, *J* = 8.85, 2.05 Hz, H-2’, H-6’), 6.90 (2H, dd, *J* = 8.90, 2.05 Hz, H-3’, H-5’), 6.39 (1H, d, *J* = 2.05 Hz, H-8), 6.18 (1H, d, *J* = 2.10 Hz, H-6); ^13^C-NMR (MeOD, 125 MHz) δ ppm: 175.9 (C=O), 164.2 (C-7), 161.1 (C-5), 159.1 (C-4’), 156.8 (C-9), 146.6 (C-2), 135.7 (C-3), 129.3 (C-2’, C-6’), 122.3 (C-1’), 114.9 (C-3’, C-5’), 103.1 (C-10), 97.9 (C-6), 93.1 (C-8) [27].

*Taxifolin* (**8**). Pale yellow crystals, wt: 4.5 mg (acetone); ^1^H-NMR (acetone-*d*_6_, 300 MHz) δ ppm: 7.07 (d, 2H, *J* = 1.92 Hz, H-2’), 6.93 (dd, 1H, *J* = 2.01, 8.13 Hz, H-6’), 6.87 (d, 1H, *J* = 8.13 Hz, H-5’), 5.96 (d, 1H, *J* = 2.10 Hz, H-6), 5.95 (d, 1H, *J* = 2.10 Hz, H-8) 5.03 (d, 1H, *J* = 11.43 Hz, H-2), 4.62 (d, 1H, *J* = 11.40 Hz, H-3); ^13^C-NMR (Acetone-*d*_6_, 75 MHz) δ ppm: 197.3 (C-4), 167.2 (C-7), 164.1 (C-5), 163.2 (C-9), 145.8 (C-4’), 145.0 (C-3’), 128.8 (C-1’), 119.6 (C-6’), 114.9 (C-2’, C-5’), 100.3 (C-10), 96.2 (C-6), 95.14 (C-8), 83.6 (C-2), 72.2 (C-3) [28].

*Loganin* (**9**). Colourless oil, wt: 2.8 mg (MeOH). MS *m*/*z* = 408.2030 [M + H_2_O]^+^, C_17_H_26_O_10_; UV (MeOH) λ_max_ nm: 295, 330; IR υ_max_ cm^−1^: 3368, 1655, 1420, 1449, 669; ^1^H-NMR (MeOD, 600 MHz) δ ppm: 7.41 (1H, *s*, H-3), 5.29 (1H, d, *J* = 4.50 Hz, H-1), 4.67 (1H, d, *J* = 7.92 Hz, H-1’), 4.06 (1H, m, *J* = 4.44 Hz, H-7), 3.92 (1H, dd, *J* = 11.91, 1.74 Hz, H-6’ax), 3.70 (3H, s, OCH_3_), 3.69 (1H, dd, *J* = 11.94, 5.70 Hz, H-6’eq), 3.39 (1H, m, H-3’), 3.32 (1H, m, H-5’), 3.31 (1H, m, H-4’), 3.22 (1H, m, H-2’), 3.13 (1H, m, H-5), 2.25 (1H, m, H-6eq), 2.04 (1H, m, H-9), 1.89 (1H, m, H-8), 1.64 (1H, m, H-6ax), 1.11 (3H, d, *J* = 6.96 Hz, CH_3_-10); ^13^C-NMR (MeOD, 150 MHz) δ ppm: 168.2 (C=O), 150.7 (C-3), 112.6 (C-4), 98.7 (C-1’), 96.3 (C-1), 70.2 (C-4’), 76.6 (C-3’), 73.7 (C-7), 73.3 (C-2’), 77.0 (C-5’), 61.4 (C-6’), 50.3 (C-13OCH_3_), 45.1 (C-9), 41.3 (C-6), 40.8 (C-8), 30.7 (C-5), 12.0 (C-10) [29].

*β-sitosterol* (**10**). Transparent needles, wt: 4.5 mg (chloroform), mp 135 °C (mp lit. 136–138 °C). C_29_H_50_O; IR (KBr) υ_max_ cm^−1^: 3426 (OH), 2950 (C-H), 1635 (C=C); ^1^H-NMR (CDCl_3_, 300 MHz) δ ppm : 5.36 (1H, d, *J* = 5.1 Hz, H-6), 3.53 (1H, m, H-3), 2.24 (1H, m, H-4), 2.00 (2H, m, H-7, H-4), 1.83 (1H, m, H-25), 1.68 (4H, m, H-7, H-20, H-15, H-16), 1.50 (6H, m, H-2, H-12, H-11, H-28, H-24, H-17), 1.42 (2H, m, H-9, H-14), 1.33 (5H, m, H-1, H-15, H-16, H-12, H-2), 1.23 (5H, m, H-11, H-22, H-23, H-1, H-8), 1.02 (3H, s, H_3_-19), 0.93 (3H, d, *J*= 6.02 Hz, H_3_-21), 0.87 (3H, t, *J* = 6.01 Hz, H_3_-29), 0.81 (3H, d, *J* = 6.62 Hz, H_3_-26), 0.69 (3H, s, H_3_-18); ^13^C-NMR (CDCl_3_, 75 MHz) δ ppm: 140.8 (C-5), 121.7 (C-6), 71.8 (C-3), 56.8 (C-14), 56.0 (C-17), 50.1 (C-9), 45.8 (C-24), 42.3 (C-13), 42.3 (C-4), 39.8 (C-12), 37.2 (C-1), 36.5 (C-10), 36.1 (C-20), 33.9 (C-22), 31.9 (C-7), 31.9 (C-8), 31.6 (C-2), 29.1 (C-25), 28.2 (C-16), 26.1 (C-23), 24.3 (C-15), 23.1 (C-28), 21.1 (C-11), 19.8 (C-26), 19.4 (C-19), 19.0 (C-27), 18.8 (C-21), 12.0 (C-29), 11.9 (C-18) [30].

### 3.5. α-Glucosidase Inhibitory Assay

The method of α-glucosidase inhibitory assay was adopted from Ahmad *et al.* [12] with minor modification. The plant samples (extract/fraction/compound) were prepared in 5% DMSO at a concentration of 1000 μg/mL. A series of dilutions for each sample were prepared to give concentrations of 1000, 500, 250, 125, 62.5 and 31.25 μg/mL. Then, about 10 μL of sample, 20 μL of α-glucosidase enzyme solution (type 1 from Baker’s yeast), phosphate buffer saline (40 μL at pH 6.5) and deionized water (20 μL) were mixed in a 96-well plate. The mixture was incubated at 37 °C. After 10 min, 10 μL of 20 mM *p*-nitrophenyl-α-D-glucopyranoside solution was added into the mixture and absorbance at time 0 min was measured by the spectrophotometer (λ = 405 nm). Then, the reaction mixture was incubated at 37 °C for 30 min and the absorbance was measured. For negative control, the sample was replaced with 5% DMSO and acarbose was used as positive control. Experiments were performed in triplicates with three independent experiments. The percentage inhibitory activity was calculated using the following equation:

(1) $$\text{Inhibition }\left(\%\right)=\frac{\left(A_{30\text{ min}}–A_{0\text{ min}}\right)\text{ control }–\;\left(A_{30\text{ min}}\;–\;A_{0\text{ min}}\right)\text{ sample }\times \;100}{\left(A_{30\text{ min}}–A_{0\text{ min}}\right)\text{ control}}$$

where A is the absorbance of mixture measured at 405 nm.

## 4. Conclusions

A phytochemical study of the methanolic stem extract of Malaysian *Uncaria cordata* var. *ferruginea* has led to the isolation of ten non-alkaloid constituents from four different classes of compounds. In the evaluation of the *in vitro* α-glucosidase inhibitory activities of this species, the methanolic stem extract and the acetone fraction both exhibited high percentages of α-glucosidase inhibition of 87.7% and 89.2%, at 1 mg/mL, respectively, while the DCM fraction demonstrated moderate inhibition (75.3%) with IC_50_ values of 102, 360 and 200 μg/mL, respectively, compared to acarbose. 2,4-DHBA and quercetin isolated from the active acetone fraction showed strong *in vitro* α-glucosidase inhibition with IC_50_ values of 549 μg/mL (or 3.56 mM) and 556 μg/mL (or 1.84 mM) respectively, against acarbose (IC_50_ 580 μg/mL or 0.89 mM). Based on the literature, the untested compounds isolated from this fraction may contribute significantly to the strong activity observed for the acetone fraction. However, loganin and scopoletin (isolated from the DCM fraction) showed only weak α-glucosidase inhibitory activities of 44.9% and 34.5%, respectively, which is supported by the weak activities of iridoid glycosides and coumarins reported in the literature. This is the first report of the isolation of 2-hydroxybenzoic acid, 2,4-dihydroxybenzoic acid and loganin from the genus and the first report of the α-glucosidase inhibitory potential of 2,4-dihydroxybenzoic acid.

## Figures and Tables

**Table 1:** α-Glucosidase inhibitory activity of stem extract, fractions and compounds from *Uncaria cordata* var*. ferruginea*.

Samples	α-Glucosidase Inhibitory * (%)	IC_50_ (μg/mL)	IC_50_ (mM)
Stems	87.7 ± 2.3	102	-
Fractions			
DCM	75.3 ± 2.4	360	-
Acetone	89.2 ± 3.8	200	-
Acarbose	90.9 ± 2.7	580	0.89
Compounds			
2,4-DHBA	78.8 ± 0.4	549	3.56
Quercetin	84.5 ± 0.6	556	1.84
Scopoletin	34.5 ± 0.4	NA	-
Loganin	44.9 ± 0.7	NA	-

* Concentration: 1 mg/mL; Values are Means ± SD, *n* = 3, NA = Not Applicable; Acarbose used as positive control.
